# Plasmonic Cu_2−x_Se Mediated Colorimetric/Photothermal Dual-Readout Detection of Glutathione

**DOI:** 10.3390/nano13111787

**Published:** 2023-06-01

**Authors:** Guojuan Yan, Huanhuan Ni, Xiaoxiao Li, Xiaolan Qi, Xi Yang, Hongyan Zou

**Affiliations:** 1 Key Laboratory of Endemic and Ethnic Diseases, Ministry of Education, Guizhou Medical University, Guiyang 550004, China; ygj792989689@email.swu.edu.cn (G.Y.); xiaolan76@163.com (X.Q.); 2 College of Pharmaceutical Sciences, Southwest University, Chongqing 400715, China; nhh1998@email.swu.edu.cn (H.N.); lxx314@email.swu.edu.cn (X.L.); 3 Department of Basic Medical Science, Guiyang Healthcare Vocational University, Guiyang 550081, China

**Keywords:** plasmonic nanomaterials, LSPR, copper selenide, GSH

## Abstract

Plasmonic nanomaterials have attracted great attention in the field of catalysis and sensing for their outstanding electrical and optical properties. Here, a representative type of nonstoichiometric Cu_2−x_Se nanoparticles with typical near-infrared (NIR) localized surface plasma resonance (LSPR) properties originating from their copper deficiency was applied to catalyze the oxidation of colorless TMB into their blue product in the presence of H_2_O_2_, indicating they had good peroxidase-like activity. However, glutathione (GSH) inhibited the catalytic oxidation of TMB, as it can consume the reactive oxygen species. Meanwhile, it can induce the reduction of Cu(II) in Cu_2−x_Se, resulting in a decrease in the degree of copper deficiency, which can lead to a reduction in the LSPR. Therefore, the catalytic ability and photothermal responses of Cu_2−x_Se were decreased. Thus, in our work, a colorimetric/photothermal dual-readout array was developed for the detection of GSH. The linear calibration for GSH concentration was in the range of 1–50 μM with the LOD as 0.13 μM and 50–800 μM with the LOD as 39.27 μM. To evaluate the practicability of the assay, tomatoes and cucumbers were selected as real samples, and good recoveries indicated that the developed assay had great potential in real applications.

## 1. Introduction

Doped semiconductor nanocrystals with a dynamic manipulation of carrier densities have attracted great attention in the field of sensing, catalysis and photonics technologies [1,2,3]. They show plasmonic optical responses similar to the localized surface plasmon resonances (LSPR) of noble metals, such as Ag and Au nanomaterials [4,5]. However, their plasmonic spectroscopic signature tuning can be achieved by modulating their doping which is not easily realized by metal nanoparticles [6]. Recently, research has been focused on semiconductor nanocrystals based on earth-abundant transition metals such as copper for their low cost [7,8]. Nonstoichiometric copper selenide nanoparticles (Cu_2−x_Se) are a typical self-doped representative with a high carrier (holes) concentration leading to the evolution of NIR LSPR [9,10]. The Cu(I)/Cu(II) always coexists in these nanocrystals (NCs), acting as a source of optically active holes, and the optical properties can be adjusted effectively by the Cu-defect, exhibiting a blueshift or redshift in the absorption spectrum located in the NIR [11,12]. Many reports have shown that their LSPR tunability in a broad wavelength range can be controlled either by developing synthesis strategies or performing red–ox reactions in the post-synthesis [13,14,15,16]. It has been proved that the stoichiometric form Cu_2_Se does not provide plasmon resonances, while its nonstoichiometric composition Cu_2−x_Se induced a localized surface plasmon extinction band in the NIR for the introduction of Cu(II) [17,18]. This phenomenon was confirmed by Dorfs et al., who showed that the addition of an excess of Cu^+^ ions could result in a reduction in the NIR LSPR band, but the addition of an oxidizer Ce(IV) could enhance the plasmon band due to the increase in copper vacancies [19].

It is worth mentioning that high copper vacancies can make Cu_2−x_Se good electron donors and acceptors; thus, they have the good catalytic ability by facilitating electron transfer [20,21]. Our group demonstrated that copper vacancies acted as reactive sites for the enhancement of chemiluminescence (CL) in the luminol–H_2_O_2_ system [22]. Furthermore, the coexistence of Cu(I)/Cu(II) in the Cu_2−x_Se nanocrystals was similar to the Fenton reagent [23,24,25,26,27]. Thus, they were proved to exhibit a peroxidase-like activity which could accelerate the oxidation of colorless 3,3′,5,5′-tetramethylbenzidine (TMB) to its blue product (oxidized TMB, OxTMB) with H_2_O_2_. Based on the catalytic activity of Cu_2−x_Se, some colorimetric assays for measuring various targets have been developed [28,29,30].

Compared with photoluminescence, photothermal responses mainly result from local temperature changes, which lead to a high sensitivity because of the low background noise [31,32]. Hessel et al. proved that Cu_2−x_Se nanocrystals exhibited an intense NIR absorbance peak and exhibited marked photoinduced heating as Au nanorods and Au nanoshells excited by a NIR laser [33]. Due to the high and stable photothermal conversion, low toxicity, and easy preparation, Cu_2−x_Se nanomaterials have been used as promising photothermal agents in photothermal therapy and sensing [34,35,36,37,38,39]. The adjustability of the photothermal efficiency due to their dynamic LSPR properties made a photothermal signal of Cu_2−x_Se nanomaterials available for qualitative analysis [40].

Glutathione (GSH), as a typical biological thiol, performs many physiological functions in the biological systems [41,42,43]. However, they can be oxidized to GSSG by the reactive oxygen species (ROS) and induced by aging or diseases [44,45]. Thus, GSH is commonly used in various medicinal and functional foods to serve as an anti-aging and immunity-enhancing component [46]. However, an overdose of GSH can result in many diseases, including Alzheimer’s disease, liver damage and cancer et al. [47]. It strongly suggests that a GSH balance should be maintained. Therefore, an accurate GSH quantification in medicine and foods is essential to provide a reference for the daily GSH supplement for disease prevention and treatment. Currently, the reported assays for GSH include fluorescence [48], colorimetric [49], electrochemiluminescence [50], etc.

**Scheme 1:**
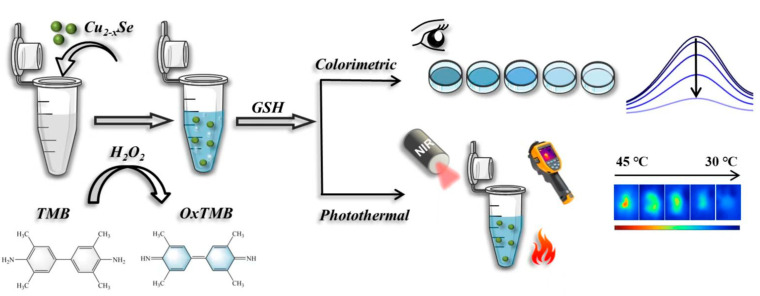
Schematic representation of the detection procedure for GSH.

To meet the growing demand for sensing technology, the integration of two sensing signals in dual-mode sensing technology not only reduces assumptions but also increases the linear range of detection and application flexibility [51]. Based on the above consideration, the Cu_2−x_Se nanoparticles in which Cu(I)/Cu(II) coexisted, acting as a source of optically active holes, were used to catalyze the oxidation of colorless TMB into its blue product in the presence of H_2_O_2_. In the catalytic process, the Cu_2−x_Se nanoparticles showed peroxidase-like activity. In the presence of GSH, the peroxidase-like activity of Cu_2−x_Se was inhibited, and its NIR LSPR was decreased. Thus, a colorimetric/photothermal dual-readout array based on the copper deficiency modulation of Cu_2−x_Se was developed for the detection of GSH (Scheme 1).

## 2. Materials and Methods

### 2.1. Materials and Instrumentation

Selenium dioxide (SeO_2_), Poly (sodium-styrennesulfonate, PSS), and 3,3′,5,5′-Tetramethylbenzidine dihydrochloride (TMB·2HCl) were supplied by Macklin Biochemical Co., Ltd. (Shanghai, China). L-Glutathione reduced (GSH) were obtained from Sigma-Aldrich (Shanghai, China). Copper sulfate (CuSO_4_·5H_2_O) and GSH array kits were supplied by Sinopharm Chemical Reagent Co., Ltd. (Shanghai, China). Ascorbic acid (Vc) and H_2_O_2_ were purchased from Chengdu Kelong Chemical Co., Ltd., (Chengdu, China).

The absorption of samples was acquired by a UV-3600 spectrophotometer (Shimadzu, Tokyo, Japan). The X-ray photoelectron spectroscopy (XPS) analyzer (Escalab 250Xi, Thermo, Waltham, MA, USA) and transmission electron microscopy (TEM) analyzer (F200X, FEI Company, Hillsboro, OR, USA) was used for the nanoprobe morphological characterization. The Electron Paramagnetic Resonance (EPR) was used for ·OH determination (A300-10/12, Bruker, Bremen, Germany).

### 2.2. Synthesis of Cu_2−x_Se NPs

The Cu_2−x_Se NPs were synthesized by a hydrothermal method [22]. In total, 0.8 mL 5 mg/mL PSS, 0.2 mL 0.05 M SeO_2_ and 15 mg/mL Vc were mixed with 5.6 mL H_2_O in a round-bottom flask for 10 min at 30 °C. A mixture of 0.4 mL 0.1 M CuSO_4_·5H_2_O and 0.8 mL 15 mg/mL Vc was added and stirred for 30 min, then, the temperature was raised to 45 °C and continued to be stirred at room temperature for 10 h until a green solution was obtained. The resulting mixture was allowed to react under vigorous stirring at room temperature for 10 h until a green solution was obtained and purified through a 10 kDa dialysis membrane for 1 day with 6 changes in the distilled water. After the dialysis, the solution was centrifuged at 10,000 rpm for 10 min and resuspended with an equal volume of water [22].

### 2.3. Analytical Process for GSH Detection

A total of 25 μg/mLCu_2−x_Se 7.5 mM H_2_O_2_, 1.5 mM TMB and different concentrations of GSH (0–800 μM) were added to the PBS solution with pH6.5, which reacted at room temperature for 30 min for subsequent detection. For the colorimetric detection, its absorbance value at the characteristic absorption peak of OxTMB (650 nm) was recorded (*A* or *A*_0_). The Δ*A* = *A*_0_ − *A* was calculated, and the GSH concentration was quantitatively analyzed according to the Δ*A*/*A*_0_.

For the photothermal detection, all solutions were exposed to a 1064 nm laser (1.0 W/cm^2^, 5 min), and the temperature and photothermal images were recorded by a thermometer and infrared thermal imager.

### 2.4. GSH Detection in Real Samples

Tomatoes and cucumbers were selected as real samples to evaluate the practicability of the array by the standard recovery method. The tomatoes and cucumbers were washed with pure water and dried naturally. Then, they were crushed with a juicer and centrifuged 3–4 times (10,000 rpm, 5 min), while the upper supernatant was filtered with a 0.22 μm filter membrane for further detection.

Briefly, 100 μL of the diluted samples and certain concentrations of GSH (30.0 μM) were added to the above detection system (25 μg/mLCu_2−x_Se, 7.5 mM H_2_O_2_, 1.5 Mm TMB, pH 6.5) and reacted for 30 min. The absorbance value at the characteristic absorption peak of OxTMB (650 nm) was recorded.

## 3. Results and Discussion

### 3.1. Characterization of Cu_2−x_Se NPs

**Figure 1:**
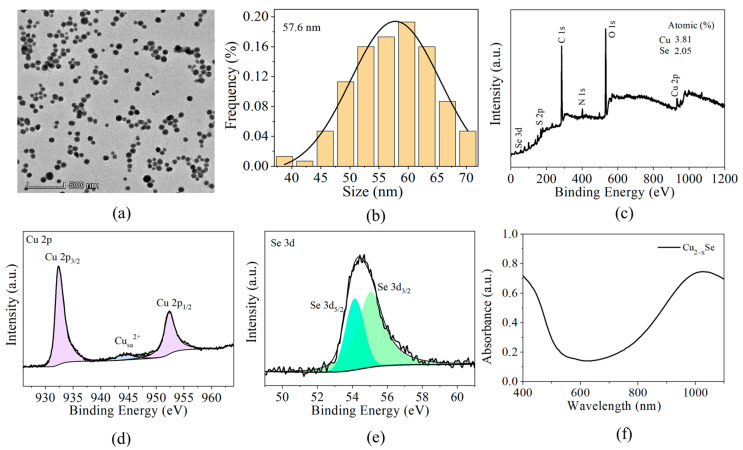
(**a**) The TEM images (**b**) Particle size distribution histograms (One hundred and fifty nanoparticles were counted). (**c**) Full-scan XPS profiles of Cu_2−x_Se NPs (**d**) High resolution XPS spectra for Cu 2p (**e**) High resolution XPS spectra for Se 3d. (**f**) The absorption spectra of Cu_2−x_Se.

The nonstoichiometric Cu_2−x_Se nanoparticles were synthesized using a hydrothermal method. Their morphologies and compositions were characterized by TEM and XPS. The TEM images displayed how the prepared Cu_2−x_Se were uniformly distributed with quasi-spherical nanoparticles at a diameter of 57.6 nm (Figure 1a,b). As shown in Figure 1c, the full-scan XPS profiles confirmed that the nanoparticles contained Cu, Se and S elements, where S came from PSS. The ratio of Cu:Se was 3.81:2.05, which confirmed the formation of Cu_2−x_Se with x = 0.14 (Cu_1.86_Se). The high-resolution spectra showed a peak at 54.2 eV for Se 3d, which demonstrated the existence of Se^2−^, and the Cu 2p_3/2_ (932.2 eV), Cu 2p_1/2_ (952.0 eV) and a satellite binding energy peak (940–950 eV) was ascribed to the co-existence of Cu(I) and Cu(II) (Figure 1d,e) [52,53]. The as-prepared Cu_2−x_Se were suspended in water for an extinction measurement via UV-vis-NIR spectroscopy. There was a strong LSPR band at 1025 nm, which was assigned to a high copper vacancy, and a shoulder absorption band at about 400–500 nm, which was ascribed to the direct band gap (Figure 1f). Compared to the metal nanoparticles with narrowband plasmonic resonance, which only allowed the utilization of a small portion of visible light, the Cu_2−x_Se with broadband NIR absorption was beneficial for enhancing the utilization efficiency of sunlight energy and also avoiding the wavelength limitation for photothermal sensing/imaging and therapy.

### 3.2. Peroxidase-Mimicking Activity Inhibition of Cu_2−x_Se by GSH

**Figure 2:**
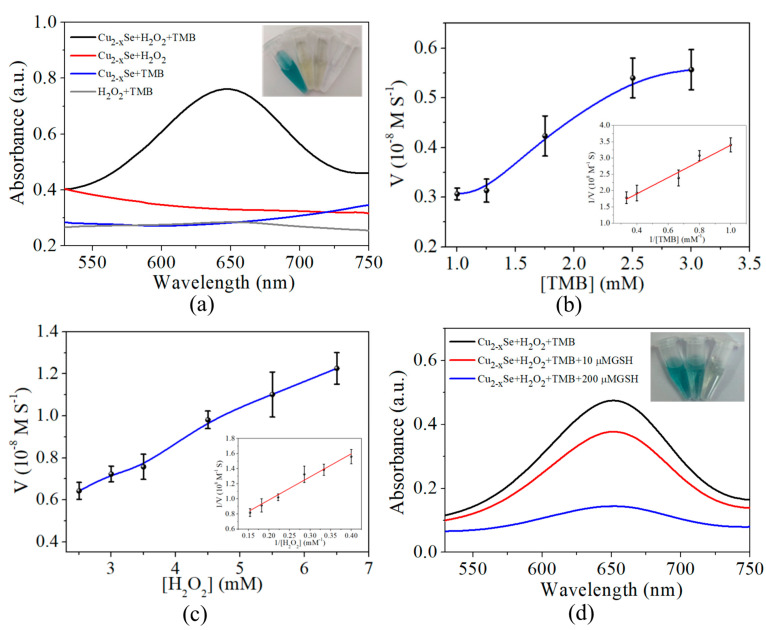
The absorption spectra of Cu_2−x_Se-H_2_O_2_-TMB system under different conditions (inset: the solutions of TMB) (**a**). Steady-state kinetic assay of the reaction speed with varied concentrations of (**b**) TMB and (**c**) H_2_O_2_ (the insets were double reciprocal curves of initial reaction rate with concentrations of substrate). (**d**) The system interacting with GSH (the solutions of TMB).

The common substrate of peroxidase, TMB, was utilized to evaluate the catalytic activity of Cu_2−x_Se. As shown in Figure 2a and Figure S1, only in the coexistence of H_2_O_2_ and Cu_2−x_Se, the colorless TMB could be oxidated as its blue derivative OxTMB, with a typical absorption peak at 650 nm. The steady-state kinetics behavior was studied at room temperature, and the catalytic reaction kinetics of Cu_2−x_Se nanoparticles followed the typical Michaelis-Menten model, which was consistent with the kinetic characteristics of horseradish peroxidase, HRP, indirectly indicated the peroxidase-mimicking activity of Cu_2−x_Se nanoparticles (Figure 2b,c). Additionally, the calculated kinetic parameters (Table S1) and the *K*m values towards H_2_O_2_ and TMB of Cu_2−x_Se were slightly lower than those of natural HRP. In order to further investigate the stability of Cu_2−x_Se as catalysts, the changes in the enzyme activity of Cu_2−x_Se nanoparticles were tested at different pH and temperatures (Figure S2). It was found that Cu_2−x_Se nanoparticles could have good enzyme activity in a wide range of pH and temperature, which was also an important advantage for further application.

However, the absorbance at 650 nm declined, and the visible color was changed from blue to colorless with the addition of GSH, suggesting that GSH could dramatically inhibit the catalytic process of Cu_2−x_Se (Figure 2d). Meanwhile, the change in the absorption intensity at 650 nm varied with the GSH concentration. On the basis of the above phenomenon, a colorimetric method for the quantitative detection of GSH was designed by monitoring the changes in absorbance at 650 nm.

### 3.3. Optimization of Experimental Conditions

The optimal experimental conditions were investigated by the univariate method, including pH, TMB concentration, Cu_2−x_Se concentration and H_2_O_2_ concentration. As shown in Figure S3a, the PBS buffer with a different pH was added to the system. With the increase in the pH value, the change in Δ*A*/*A*_0_ first increased and then decreased, and the maximum value was obtained when the pH was 6.5. Therefore, pH 6.5 was selected as the optimum value. Then, with the fixed pH at 6.5, different concentrations of TMB, Cu_2−x_Se and H_2_O_2_ were investigated in turn. As shown in Figure S3b–d, the maximum Δ*A*/*A*_0_ was reached when the concentration of TMB was 1.5 mM, Cu_2−x_Se was 25 μg/ML, and H_2_O_2_ was 7.5 mM. To obtain a highly sensitive response for the quantitative detection of GSH, the final optimized conditions were as follows: Ph 6.5, TMB: 1.5 mM, Cu_2−x_Se NPs: 25 μg/mL and H_2_O_2_: 7.5 mM.

### 3.4. Performance of GSH Colorimetric Detection

**Figure 3:**
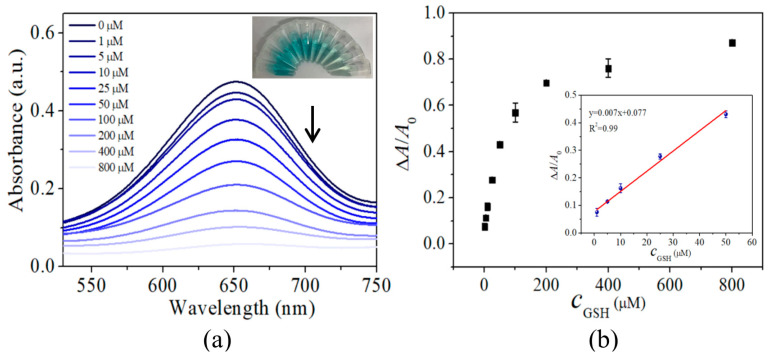
(**a**) Absorption spectra of Cu_2−x_Se-H_2_O_2_-TMB incubated with different concentrations of GSH (0, 1, 5, 10, 25, 50, 100, 200, 400 and 800 μM). (**b**) The change in the absorbance with the increase in GSH concentration (the inset showed the linear relationship between the absorbance and concentrations of GSH within the range of 1–50 μM).

Under the optimum reaction conditions, different concentrations of GSH were added to the sensing systems. As shown in Figure 3a, with the increase in the GSH concentration (0 μM–800 μM), the absorbance of OxTMB at 650 nm gradually decreased, and the color of the solution changed from blue to light blue and then to colorless. There is a good linear relationship between the change in the absorbance signal and the GSH concentration in the range of 1–50 μM (Figure 3b). A linear equation could be expressed as Δ*A*/*A*_0_ = 0.007*c* + 0.077 (*R*^2^ = 0.99), where *c* was the concentration of GSH. Additionally, the LOD (3*σ*/*k*) was 0.13 μM.

The interferences were investigated to evaluate the anti-interference capability of this sensing platform. As shown in Figure S4, no changes in the detection signal were observed which was compared to those of the control in the presence of GSH when the common interferences (K^+^, Na^+^, Ca^2+^, Mg^2+^, glucose, glutamine, furfural, lysine, proline, valine and Vc) were added. It demonstrated that this method for GSH would not be affected by other interferences, and this established detection assay possessed good selectivity and could further be applied for the quantitative analysis of GSH in actual samples.

### 3.5. Detection of GSH in Real Samples

The feasibility of the proposed method for real sample analyses was investigated by determining the GSH contents in tomatoes and cucumbers. As shown in Table 1, the contents of the tomatoes and cucumbers determined by this method were comparable with the GSH kit testing. Additionally, the quantitative spike recoveries were determined to be 95.6–106.3%, indicating the practicability of the proposed method in the food samples.

### 3.6. Photothermal Detection of GSH

**Figure 4:**
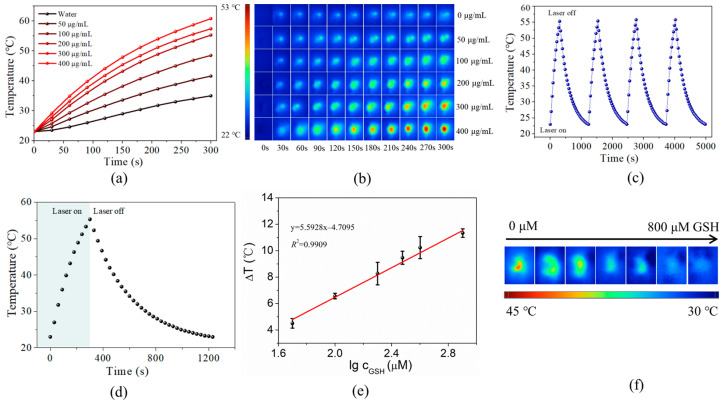
(**a**) Temperature change curves of Cu_2−x_Se with different concentrations under laser irradiation (1064 nm, 1.0 W/cm^2^). (**b**) The corresponding photothermal images (The recording time was 0, 30, 60, 90, 120, 150, 180, 210, 240, 270 and 300s, and the concentration of Cu_2−x_Se was 0, 50, 100, 200, 300 and 400 μg/mL). (**c**) Temperature curves of Cu_2−x_Se for four laser on/off cycles under laser irradiation. (**d**) Temperature profile of Cu_2−x_Se under laser irradiation. (**e**) The linear relationship between the Δ*T* and concentrations of GSH within the range of 50 μM–800 μM. (**f**) The photothermal images of Cu_2−x_Se-H_2_O_2_-TMB incubated with different concentrations of GSH (0, 50, 100, 200, 300, 400 and 800 μM) under laser irradiation at 5 min.

Nonstoichiometric Cu_2−x_Se with Cu deficiencies exhibited NIR absorption, and the high molar extinction coefficients 3.18 × 10^8^ M^−1^ cm^−1^ at 1058 nm were measured (Figure S5), which were much higher than those of organic dyes and semiconductor quantum dots (Table S2). A significant amount of heat was observed when the NIR plasmon resonance was optically excited, and the light energy was converted into heat energy [54]. The NIR absorbance peak of Cu_2−x_Se has been proven to be a surface plasmon resonance with high vacancy concentrations and was widely used in the field of photothermal therapy [55] and photothermal immunoassay [56], etc. As shown in Figure 4a, under the irradiation of a 1064 nm laser (1.0 W/cm^2^), the temperature of the Cu_2−x_Se solution increased from room temperature rapidly to over 50 °C in 5 min. In contrast, there was little change in the temperature of pure water, indicating that the light energy could be efficiently absorbed and converted into heat, which is consistent with the previous reports [57]. The clear photothermal images were recorded using an IR camera (Figure 4b). It also showed excellent photothermal stability during four cycles of on/off laser irradiation, where no decay in the temperature was observed (Figure 4c). The photothermal heat conversion efficiency of Cu_2−x_Se (η) was measured to be 23.68% (Figure 4d, Figures S6 and S7 and details in the supporting information).

However, when a certain amount of GSH was added (0, 50, 100, 200, 300, 400 and 800 μM), the temperature of the Cu_2−x_Se solution decreased, and the image point captured by the thermal imager is shown in Figure 4f. It gradually changed from red to blue, indicating that the temperature gradually decreased. There was a direct relationship between the temperature change and the concentration of GSH; the relationship curve was fitted and had a good linear relationship in the range of 50–800 μM, wherein the LOD (3*σ*/*k*) was 39.27 μM (Figure 4e). This phenomenon could also provide a sensing protocol for GSH detection.

### 3.7. Mechanisms

**Figure 5:**
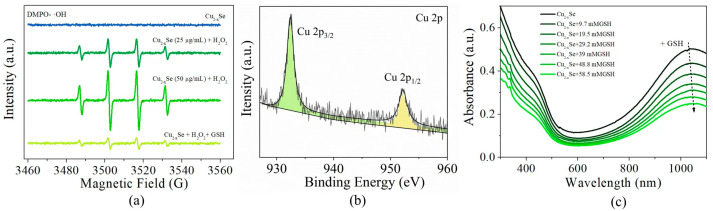
(**a**) EPR spectra in different conditions; (**b**) High resolution XPS spectra for Cu 2p after reaction GSH; (**c**) UV-Vis absorbance spectra of Cu_2−x_Se after reaction with different concentrations of GSH.

EPR spectra were used to investigate the intrinsic mechanism of the peroxidase-like activity of Cu_2−x_Se. As shown in Figure 5a, in the presence of Cu_2−x_Se, the characteristic peaks of DMPO-·OH adducts were observed in the mixtures of DMPO and H_2_O_2_, indicating the generation of ·OH in the catalytic process. In addition, the signal intensity was enhanced with the Cu_2−x_Se concentration, which revealed that the catalytic activity of Cu_2−x_Se was concentration-dependent. These results proved that the reaction mechanism between Cu_2−x_Se and H_2_O_2_ could be modeled by the Fenton reaction and the release of ·OH induced the oxidation of colorimetric reaction. However, with the addition of GSH, the signal intensity of DMPO-·OH adducts was decreased, which could inhibit the peroxidase-mimicking activity.

The Cu_2−x_Se NPs were stable as there were no significant absorbance changes observed for 5 days (Figure S8). However, after the addition of GSH, the high-resolution Cu XPS characterization of Cu_2−x_Se, the copper species were basically Cu(I), indicating that the interaction between GSH and Cu_2−x_Se occurred (Figure 5b). When the Cu_2−x_Se NPs pretreated with GSH were purified and then incubated with TMB and H_2_O_2_, the absorption intensity at 650 nm was also slightly decreased compared to that in the Cu_2−x_Se-H_2_O_2_-TMB system (Figure S9). Furthermore, when the GSH was added to the Cu_2−x_Se solution, the NIR LSPR peak was red-shifted and had a decreased absorption trend (Figure 5c). Thus, the photothermal transduction efficiency was decreased as it was positively correlated with the absorption of Cu_2−x_Se NPs [31].

## 4. Conclusions

In conclusion, a type of Cu_2−x_Se nanoparticles was used as both the nano-enzymatic and photothermal reagent. Because of the high copper deficiency, Cu(I)/Cu(II) coexisted in the Cu_2−x_Se nanoparticles and acted as a source of optically active holes. GSH, as a natural hole-scavenger, captured the holes of Cu_2−x_Se, inducing the peroxidase-like activity inhibition and the decreased photothermal efficiency, which were regulated by their reduced NIR LSPR. Therefore, a Cu_2−x_Se mediated colorimetric/photothermal dual-readout sensing platform for glutathione was developed and applied in the real samples with a satisfactory result. It is worth noting that in our proposed dual-readout sensing platform, only the possibility of detection based on photothermal signals is presented, and it has not yet been applied to the simultaneous detection of the same real samples. Therefore, there is more research to be performed on how Cu_2−x_Se nanomaterials can be used to prepare a perfect dual platform.

## Figures and Tables

**Table 1:** The results of the recovery experiment for the determination of GSH content (*n* = 3).

Samples	Standard Added GSH (μM)	Detected GSH (μM)	Recovery (%)	GSH Kit
tomatoes	0	6.00 ± 0.60	/	6.41 ± 0.51
	30.0	37.92 ± 0.35	104.4–106.3	36.76 ± 1.96
cucumbers	0	13.14 ± 0.47	/	15.17 ± 0.94
	30.0	42.45 ± 1.20	95.6–101.2	43.81 ± 1.48

## Data Availability

Not applicable.

## Supplementary Materials

The following supporting information can be downloaded at: https://www.mdpi.com/article/10.3390/nano13111787/s1, Figure S1: The individual components used in the synthesis with glutathione to oxidize the TMB; Figure S2: The relative activity of Cu_2−x_Se at different pH and temperature; Figure S3: The changes of the Δ*A*/*A*_0_ with (a) different pH, (b) different concentrations of TMB, (c) different concentrations of Cu_2−x_Se, (d) different concentrations of H_2_O_2_; Figure S4: Selectivity of the sensing system for the detection of GSH; Figure S5: (a) UV-Vis absorbance spectra of Cu_2−x_Se measured at different concentration (b) Plots of absorbance vs. concentration for Cu_2−x_Se at specific wavelength (1058 nm) with linear regression curve; Figure S6: Temperature profile of H_2_O under laser irradiation (black line), and linear relationship between –lnθ and time in the cooling stage (blue line); Figure S7: The linear relationship between –lnθ and time in the cooling stage of Cu_2−x_Se; Figure S8: The absorbance of Cu_2−x_Se in 5 days; Figure S9: The absorbance of GSH pretreated Cu_2-x_Se-H_2_O_2_-TMB and Cu_2-x_Se-H_2_O_2_-TMB system. Table S1: Comparison of the kinetic constants of Cu_2−x_Se with other nanozymes and HRP; Table S2: Moalr extinction coefficients (per mol of molecules or nanocrystals) of common photoabsorbers. References [58,59,60,61,62,63] are cited in the supplementary materials.
