# Supplementary material for: Plasmonic Cu2−xSe Mediated Colorimetric/Photothermal Dual-Readout Detection of Glutathione
Source: Nanomaterials (Basel). 2023 Jun 1;13(11):1787. doi: 10.3390/nano13111787 (PMC10255549; doi:10.3390/nano13111787)
Supplement: Supplementary file 1 [file nanomaterials-13-01787-s001.zip › nanomaterials-2385263-supplementary proof.pdf]

nanomaterials

MDPI

Supplementary Materials

Plasmonic $Cu_{2-x}Se$ Mediated Colorimetric/Photothermal Dual-Readout Detection of Glutathione

Guojuan Yan [1,2], Huanhuan Ni [2], Xiaoxiao Li [2], Xiaolan Qi [1], Xi Yang [3,*] and Hongyan Zou [2,*]

1 Key Laboratory of Endemic and Ethnic Diseases, Ministry of Education, Guizhou Medical University, Guiyang 550004, China; ygj792989689@email.swu.edu.cn (G.Y.); xiaolan76@163.com (X.Q.)
2 College of Pharmaceutical Sciences, Southwest University, Chongqing 400715, China; nhh1998@email.swu.edu.cn (H.N.); lxx314@email.swu.edu.cn (X.L.)
3 Department of Basic Medical Science, Guiyang Healthcare Vocational University, Guiyang 550081, China
* Correspondence: m1890850220@163.com or yangxi@gyhvu.edu.cn (X.Y.); zhy2013@swu.edu.cn (H.Z.)

Experimental Procedures

Calculation of the molar extinction coefficient

The molar extinction coefficient of $Cu_{2-x}Se$ NPs were caculated from the Beer-Lambert law by linearly fitting the absorbances for various concentrations of nanoparticles,

$$A(\lambda)=\varepsilon LC \tag{S1}$$

Where A is the absorbance at a wavelength λ, ε is the molar extinction coefficient, L is the pathlength (1 cm), and C is the concentration of nanoparticles (mole L^{-1}). The extinction coefficient was determined by plotting the slope of each linear fit against wavelength. The C (mole L^{-1}) of $Cu_{2-x}Se$ is calcuated using the following:

$$C=\frac{C_{wt}}{M_{NP}}=\frac{C_{wt}}{\frac{4}{3}\pi R^3\rho_{Cu_{2-x}Se}\,N_A} \tag{S2}$$

C_{wt} is the weight concentration of the nanoparticles (g L^{-1}), M_{NP} is the molar weight of the nanoparticles (g mol^{-1}), R is the average radius of the nanoparticles (21.3 nm), ϱ is the densiy of the $Cu_{2-x}Se$ (5.8 g cm^{-1}), N_A is Avogadro's constant (6.022*10^{23} mol^{-1}). Above all, the molar extinction coefficient is caculated by :

$$\varepsilon=\frac{A(\lambda)}{LC}=\frac{A(\lambda)\frac{4}{3}\pi R^3\rho_{Cu_{2-x}Se}\,N_A}{LC_{wt}} \tag{S3}$$

Photothermal activity and photothermal conversion efficiency

In order to evaluate the photothermal performance of $Cu_{2-x}Se$ NPs, 200 µL $Cu_{2-x}Se$ solution with various concentrations (0, 50, 100, 200, 300, 400 µg/mL) was exposed to 1064 nm laser (1.0 W/cm^2, 5min), and the temperature variation were detected by the thermometer (LIHUAJIN, T1320), the infrared thermal images were recorded every 30 s with infrared thermal imager (Fluke Ti400, USA).

The photothermal conversion efficiency (η) of $Cu_{2-x}Se$ was calculated according to equation (2)-(4):

$$\eta=\frac{mc(T_{max}-T_{surr})-Q_{dis}}{I(1-10^{-A_{1064}})\tau_s} \tag{S4}$$

$$\tau_s=-\frac{t}{\ln(\theta)} \tag{S5}$$

$$\theta = \frac{T - T_{surr}}{T_{max} - T_{surr}} \tag{S6}$$

$$Q_{dis} = \frac{mc(T_{max\,water} - T_{surr})}{\tau_{s\,water}} \tag{S7}$$

where the m and c were the mass (0.2 g) and heat capacity (4.2 J/g) of the water, T_{max} was the maximum equilibrium temperature of $Cu_{2-x}Se$ (55.3 ℃), T_{surr} (23.0 ℃) and T represent the environment and solution temperature, I was the laser power (0.32 W), A_{1064} was the absorbance of $Cu_{2-x}Se$ solution at 1064 nm as 0.710, τ_s was the sample system time constant (232.15), Q_{dis} represents the heat loss due to absorption by the container, T_{max} and τ_s of water was 35 ℃ and 220.46.

Peroxidase-like activity

The Peroxidase-like activity assays of $Cu_{2-x}Se$ NPs were performed using TMB and H_2O_2 as substrates. 25 μg/mL $Cu_{2-x}Se$, 7.5 mM H_2O_2 and different concentrations of TMB (0.75-3.0 mM) were added to PBS solution at pH 6.5, and the change of its absorbance (650 nm) in 60 min was recorded. Similarly, changed the concentration of H_2O_2 (2.5-7.5 mM) and TMB (1.5 mM), and recorded the change of their absorbance (650 nm) under the same conditions. Enzyme kinetic parameters were calculated by Michaelis-Menten equation:

$$v_0 = \frac{v_{max}\,[S]}{K_m + [S]} \tag{S8}$$

In this equation, v_0 is the initial reaction rate of substrate conversion, V_{max} is the maximum reaction rate of substrate conversion, [S] is the substrate concentration, and K_m is the Michaelis–Menten constant.

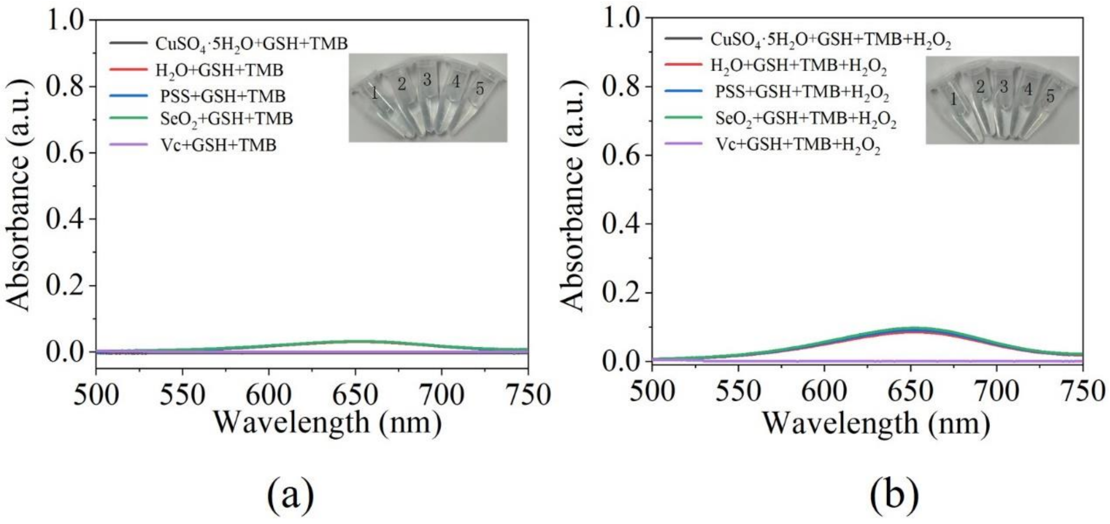

Figure S1. (**a**) The individual components used in the synthesis with glutathione to oxidize the TMB; (**b**) The individual components used in the synthesis with glutathione to oxidize the TMB with H_2O_2.

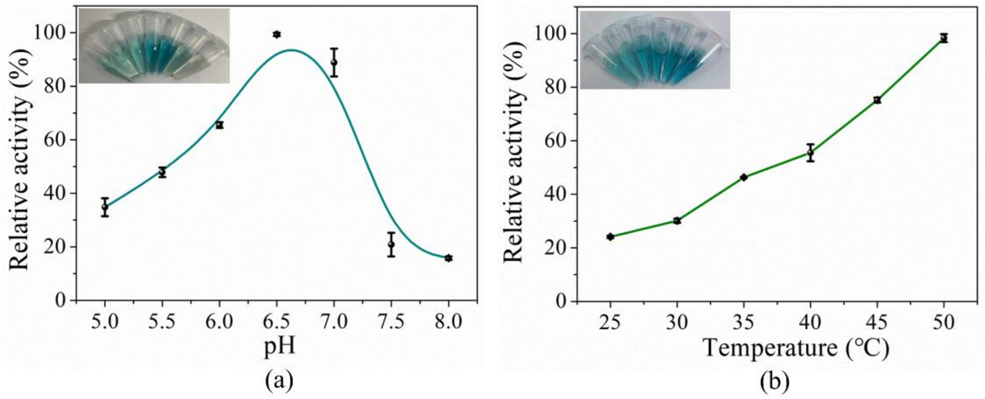

Figure S2. (**a**) The relative activity of $Cu_{2-x}Se$ at different pH (5.0, 5.5, 6.0, 6.5, 7.0, 7.5 and 8.0) and (**b**) the relative activity of $Cu_{2-x}Se$ at different temperatures (25, 30, 35, 40, 45 and 50 °C).

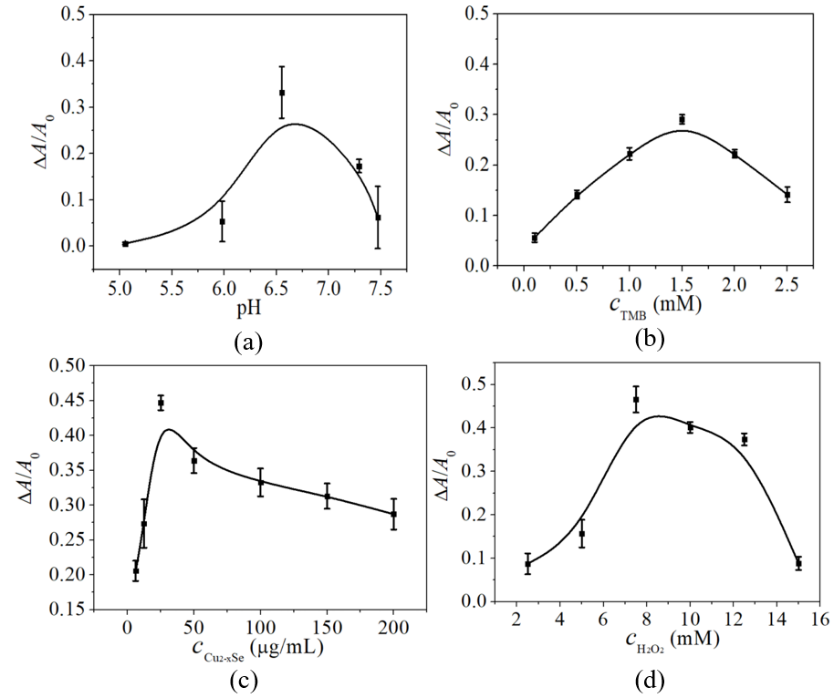

Figure S3. The changes of the $\Delta A/A_0$ with (**a**) different pH (5.0, 6.0, 6.5, 7.4 and 7.5) (**b**) different concentrations of TMB (0.1, 0.5, 1.0, 1.5, 2.0 and 2.5 mM) (**c**) different concentrations of $Cu_{2-x}Se$ (6.25, 12.5, 25, 50, 100, 150 and 200 μg/ml) (**d**) different concentrations of H_2O_2 (2.5, 5, 7.5, 10, 12.5 and 15 mM).

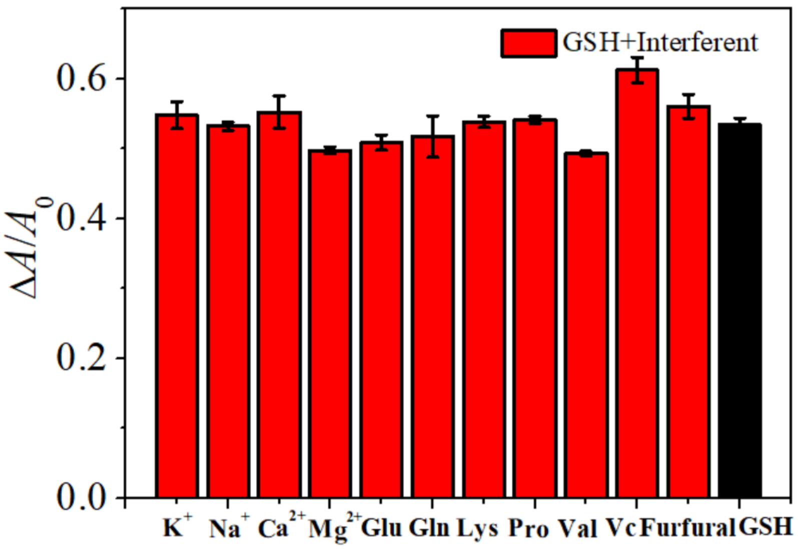

Figure S4. Selectivity of the sensing system for the detection of GSH.

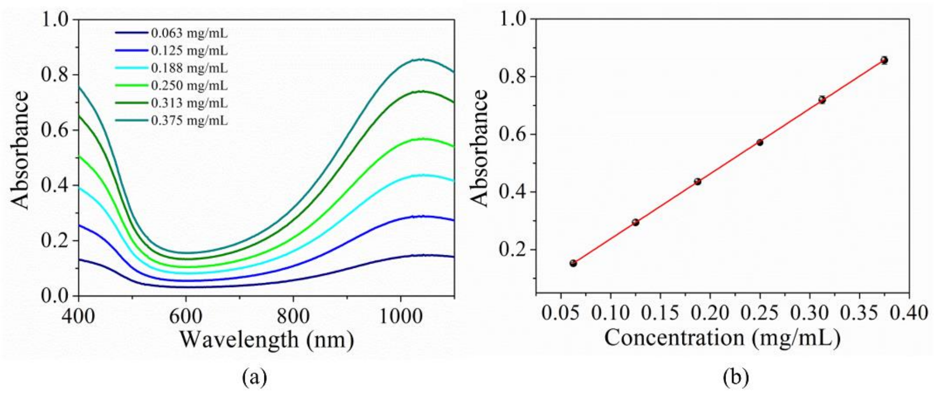

Figure S5. (**a**) UV-Vis absorbance spectra of $Cu_{2-x}Se$ measured at different concentration (**b**) Plots of absorbance vs concentration for $Cu_{2-x}Se$ at specific wavelength (1058 nm) with linear regression curve.

Table S1. Comparison of the kinetic constants of $Cu_{2-x}Se$ with other nanozymes and HRP.

Catalyst	Substrate	K_m (mM)	V_{max} (M S^{-1})	References
$Cu_{2-x}Se$	H_2O_2	8.16	2.67 x 10^{-8}	this work
	TMB	2.74	1.10 x 10^{-8}	
HRP	H_2O_2	3.7	8.7 x 10^{-8}	[1]
	TMB	0.4376	1.0 x 10^{-7}	
$Ag@Fe_3O_4$	H_2O_2	75.2	2.28 x 10^{-8}	[2]
	TMB	3.46	2.288 x 10^{-8}	

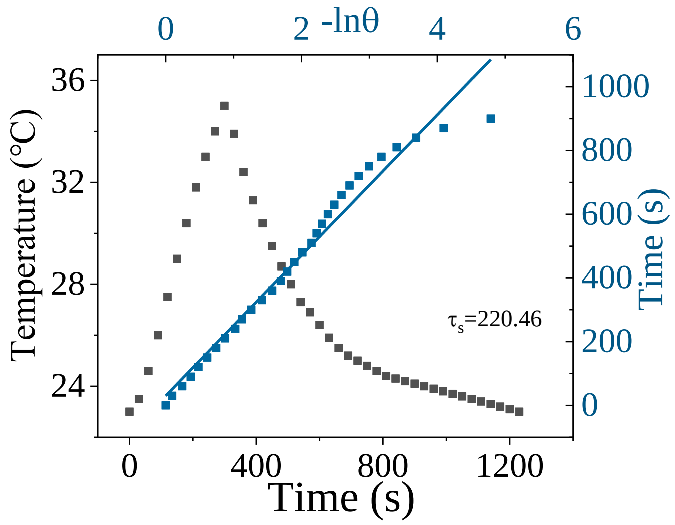

Figure S6. Temperature profile of H_2O under laser irradiation (black line), and linear relationship between –lnθ and time in the cooling stage (blue line).

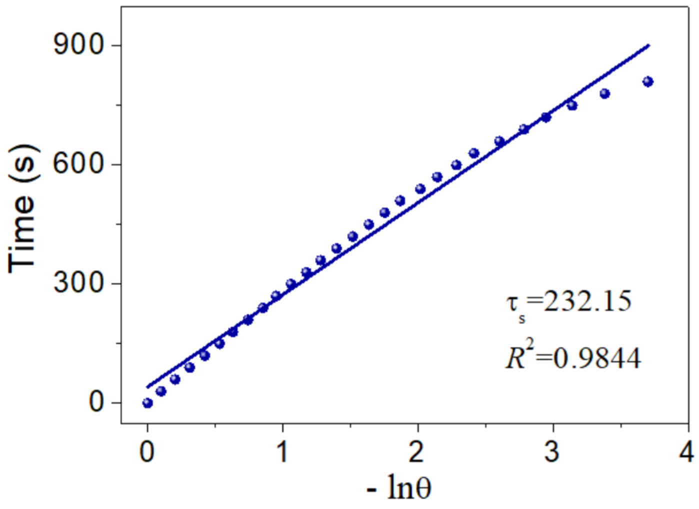

Figure S7. The linear relationship between –lnθ and time in the cooling stage of $Cu_{2-x}Se$.

Table 2. Molar extinction coefficients (per mol of molecules or nanocrystals) of common photoabsorbers.

Photoabsorber	Dimension (nm)	Molar extinction coefficient (M^{-1} cm^{-1})	Wavelength (nm)
Rhodamine 6G [3]	molecular	1.2 X 10^5	530
Malachite Green [4]	molecular	1.5 X 10^5	617
Carbon nanotubes [5]	*r*=0.6<br>*L*=150	7.9 X 10^6	808
Gold nanospheres [6]	*r*=20	~7.7 X 10^9	530

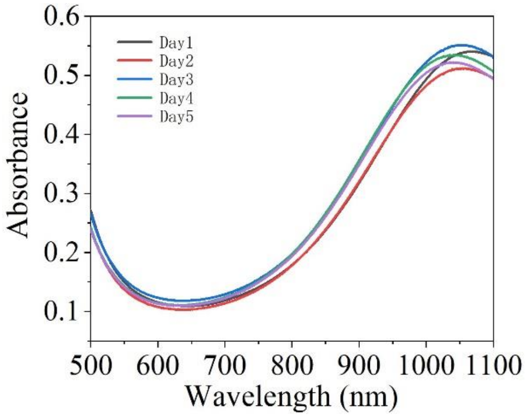

Figure S8. The absorbance of $Cu_{2-x}Se$ in 5 days.

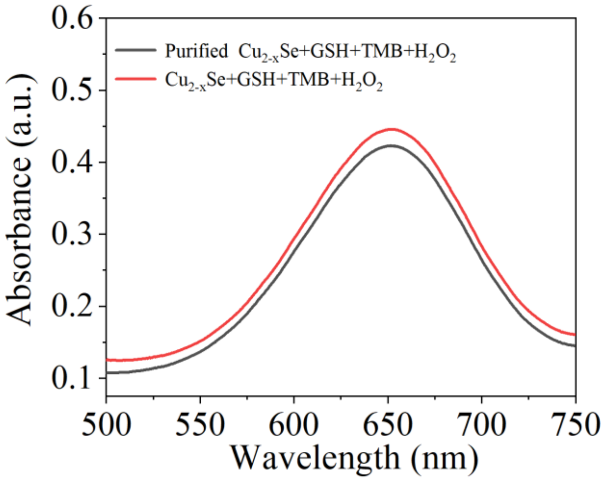

Figure S9. The absorbance of GSH pretreated $Cu_{2-x}Se$-H_2O_2-TMB and $Cu_{2-x}Se$-H_2O_2-TMB system.

References

1. B. Jiang, D. Duan, L. Gao, et al. Standardized assays for determining the catalytic activity and kinetics of peroxidase-like nanozymes[J]. Nat. Protoc., 2018, 13(7): 1506-1520.
2. J. Chen, Y. Liu, G. Zhu, et al. Ag@Fe_3O_4 nanowire: fabrication, characterization and peroxidase-like activity[J]. Crystal Research and Technology, 2014, 49(5): 309-314.
3. Du, H.; Fuh, R. C. A.; Li, J. Z.; Corkan, L. A.; Lindsey, J. S. Photochem. Photobiol. 1998, 68, 141–142.
4. Yu, W. W.; Qu, L. H.; Guo, W. Z.; Peng, X. G. Chem. Mater. 2003, 15, 2854–2860.
5. Kam, N. W. S.; O'Connell, M.; Wisdom, J. A.; Dai, H. J. Proc. Natl. Acad. Sci. U.S.A. 2005, 102, 11600–11605..
6. Jain, P. K.; Lee, K. S.; El-Sayed, I. H.; El-Sayed, M. A. J. Phys. Chem. B 2006, 110, 7238–7248.
